# O4.7. PLACENTAL GENE EXPRESSION, OBSTETRICAL HISTORY AND POLYGENIC RISK FOR SCHIZOPHRENIA

**DOI:** 10.1093/schbul/sby015.213

**Published:** 2018-04-01

**Authors:** Gianluca Ursini, Giovanna Punzi, Qiang Chen, Stefano Marenco, Joshua F Robinson, Annamaria Porcelli, Emily Hamilton, Marina Mitjans, Giancarlo Maddalena, Martin Begemann, Jan Seidel, Hidenaga Yanamori, Andrew E Jaffe, Karen F Berman, Michael F Egan, Richard Straub, Carlo Colantuoni, Giuseppe Blasi, Ryota Hashimoto, Dan Rujescu, Hannelore Ehrenreich, Alessandro Bertolino, Daniel Weinberger

**Affiliations:** 1 Lieber Institute for Brain Development; 2 National Institute of Mental Health, National Institutes of Health; 3 UCSF; 4 University of Bari ‘Aldo Moro’; 5 Max Planck Institute of Experimental Medicine, DFG Research Center for Nanoscale Microscopy and Molecular Physiology of the Brain (CNMPB); 6 Osaka University Graduate School of Medicine, Molecular Research Center for Children’s Mental Development, United Graduate School of Child Development, Osaka University; 7 Lieber Institute for Brain Development, Johns Hopkins Medical Campus; 8 Merck and Co., Inc; 9 University of Halle

## Abstract

**Background:**

Early life events influence later susceptibility to many adult diseases and may contribute to define the environmental context in which genes enhance risk for complex disorder like schizophrenia. Here we analyze the role of intrauterine and perinatal environment in modulating the association of schizophrenia with genomic risk.

**Methods:**

We evaluated whether genomic risk for schizophrenia interacts with intrauterine and perinatal complications (Early Life Complications, ELCs) on case-control status, in three independent samples of healthy subjects and patients with schizophrenia from USA (n=501), Italy (n=273) and Germany (n=919). We further analyzed the relationship between genomic risk and ELCs in two samples of only patients with schizophrenia from Germany (n=1019) and Japan (n=172). Genomic risk was measured with polygenic risk profile scores based on GWAS-significant alleles (PRS), while ELCs history was assessed with the McNeil-Sjöström Scale. We tested whether genes overlapping the schizophrenia loci interacting with ELCs are enriched in placenta and differentially expressed in placental samples from complicated pregnancies, in 8 independent placental datasets. Finally, we evaluated whether GWAS SNPs marking loci containing genes highly expressed and dynamically modulated in placenta (PlacPRS genes) drive the interaction between PRS and ELCs, and performed pathway analyses on PlacPRS genes.

**Results:**

PRS interacts with ELCs on case-control status, in the three independent samples from USA (p= p=0.004), Italy (p=0.018) and Germany (p=0.018); in each sample the variance of schizophrenia explained by PRS is multiplicatively higher in the presence of a history of ELCs compared with the absence of such events. The relationship between genomic risk and ELCs is further replicated in the two independent samples of only cases from Germany (p=0.047) and Japan (p=0.044). The gene-set based on PRS loci interacting with ELCs is highly expressed in multiple placental tissues (p<0.001) and dynamically regulated in placental samples from complicated, in comparison with normal, pregnancies (p<0.05). These differences are significantly greater in placentae from male compared with female offspring (p<10–8). The interaction between PRS and ELCs is largely driven by PlacPRS genes (p=0.002); PRS constructed from the remaining loci do not interact with ELCs (NonPlacPRS, p=0.60). Pathways and biological functions associated with NonPlacPRS genes are reminiscent of previous analyses about schizophrenia risk-genes, while PlacPRS genes implicate an orthogonal biology, with roots in the fetal/placental response to hypoxic stress.

**Discussion:**

Our data suggest that the most significant schizophrenia GWAS variants contribute to risk at least partly by converging on a developmental trajectory sensitive to ELCs and altered placental gene expression. The sex-associated effects on placental transcription suggest that the male preponderance of schizophrenia may arise from gene-environment interactions that influence placental biology. These results highlight placental health as a new public health frontier for primary prevention, particularly in high-risk males.

